# Improving oral cavity cancer diagnosis and treatment with fluorescence molecular imaging

**DOI:** 10.1111/odi.13308

**Published:** 2020-03-13

**Authors:** Jasper Vonk, Jaron Gérard de Wit, Floris Jan Voskuil, Max Johannes Hendrikus Witjes

**Affiliations:** ^1^ Department of Oral & Maxillofacial Surgery University of Groningen University Medical Center Groningen Groningen The Netherlands

**Keywords:** early diagnosis, fluorescence imaging, fluorescence‐guided surgery, molecular imaging, oral squamous cell carcinoma, surgical resection margin evaluation

## Abstract

Early diagnosis and radical surgical excision of oral squamous cell carcinomas are essential for achieving optimal treatment outcomes. To date, diagnostic tools that rely on anatomical anomalies provide limited information and resolution in clinical practice. As a result, oral cancer is often detected in an advanced stage. Also, no reliable *real‐time* intraoperative tools are readily available for the evaluation of surgical resection margins. Fluorescence imaging visualises biological processes that occur in early carcinogenesis and could, therefore, enable detection of small tumours in early stages. Furthermore, due to the high sensitivity and spatial resolution, fluorescence imaging could assist in resection margin assessment during surgery. In this review, we discuss several techniques that employ fluorescence for early diagnosis and surgical guidance in oral squamous cell carcinoma and present future perspectives on the potential of fluorescence imaging in oral cancer in the near future.

## 1 INTRODUCTION

Early diagnosis and complete tumour resection are considered to be the main pillars of oral squamous cell carcinoma (OSCC) treatment (Mitchell et al., 2018; Wong et al., 2012; Woolgar & Triantafyllou, 2005). Despite improvements in both diagnosis and treatment over the last 30 years, the five‐year survival rate of OSCC remains as low as 50% (Leoncini et al., 2018; Warnakulasuriya, 2009). The current diagnostic workup of a patient presenting with a suspicious lesion in the oral cavity involves visual inspection followed by incisional biopsy (Macey et al., 2015). Unfortunately, the human eye has limited potential to differentiate between (pre‐)malignant and benign lesions (e.g., inflammatory lesions) since their appearances can be quite similar. This might lead to a delay in diagnosis, illustrated by the fact that a substantial number of patients with OSCC present with advanced disease (stage III/IV) (Kossatz et al., 2016). To date, several methods have been pursued to aid in differentiating malignant from benign lesions, including vital staining, brush cytology and light‐based detection techniques, but all have their limitations (Macey et al., 2015). An incisional biopsy is considered the gold standard for diagnosis, but a discordance remains for up to 12% of the cases between the initial diagnosis and final histopathology after complete specimen excision (Chen, Forman, Sadow, & August, 2016). This is mainly attributed to sampling error due to the heterogeneity of the tumour (Chen et al., 2016) illustrating that early and adequate diagnosis of OSCC remains a clinical challenge.

Once a tumour lesion is correctly identified, surgery is the primary treatment modality in the majority of cases. The aim of surgical removal is to obtain a tumour‐negative resection margin of 5 mm (Helliwell & Woolgar, 2013). Multiple reports show that tumour‐positive resection margins, defined as tumour cells within 1 mm of the resection margin, significantly worsen prognosis (Smits et al., 2016; Woolgar & Triantafyllou, 2005).

Conventional imaging techniques have been proposed to assist in the diagnosis and treatment of OSCC, including computed tomography (CT), magnetic resonance imaging (MRI) and ultrasound. However, the applicability of these imaging modalities is limited in OSCC due to being expensive, lacking the necessary imaging resolution and specificity, and because the cellular processes that occur in early‐stage carcinogenesis cannot be detected (Hussain & Nguyen, 2014). Novel imaging techniques, such as fluorescence imaging (FI), might be of additional value for the diagnosis and treatment of OSCC by providing *real‐time* information with a high imaging resolution and sensitivity, thereby assisting the clinical decision‐making (de Boer et al., 2015). In this review, we focus on the current (clinically) available wide‐field FI techniques that can assist in OSCC diagnosis or surgery. Other optical imaging techniques and the emerge of optoacoustic imaging, which is highly relevant to the field, have been reviewed elsewhere (Alam et al., 2018; Attia et al., 2019; Pogue, Rosenthal, Achilefu, & Dam, 2018; Taruttis, Dam, & Ntziachristos, 2015).

## 2 FLUORESCENCE IMAGING—GENERAL PRINCIPLES

**Figure 1:**
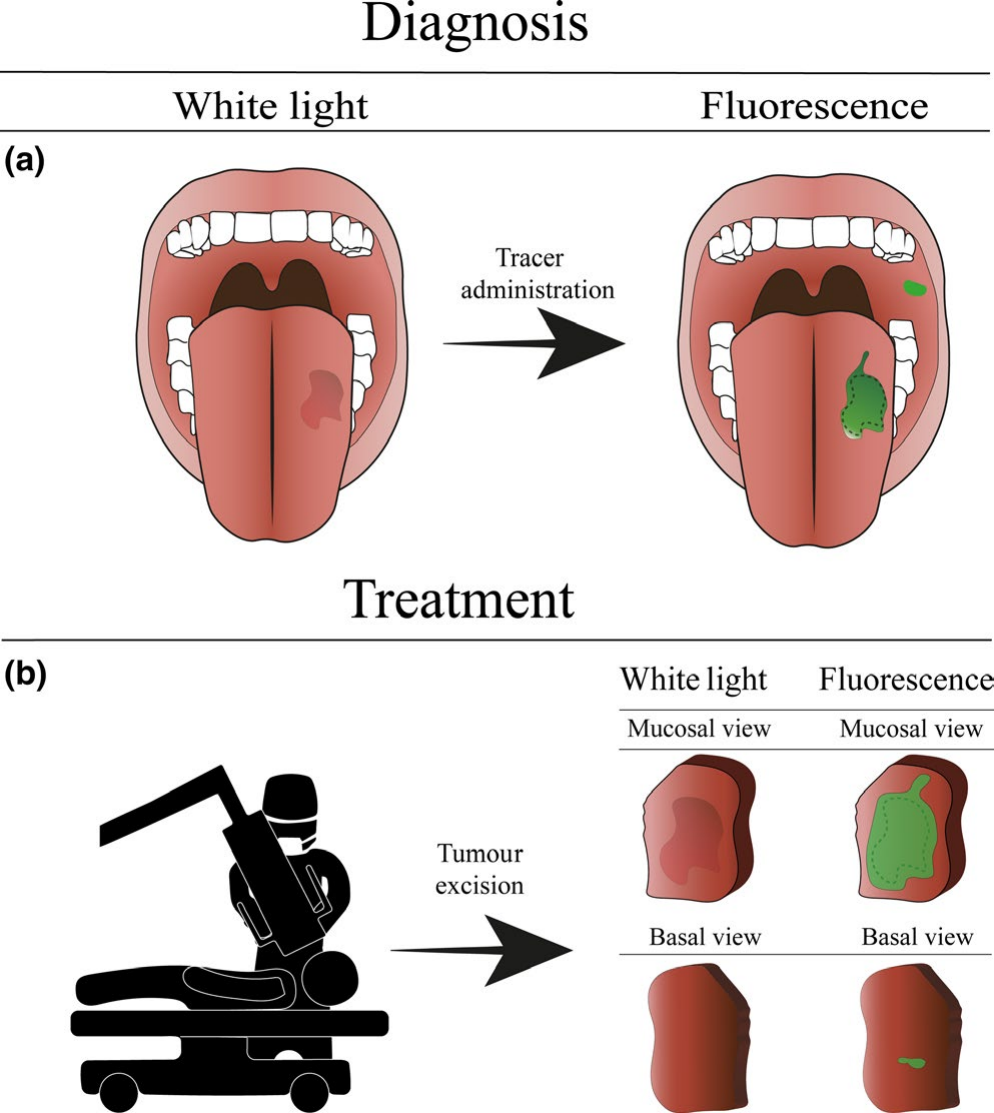
Schematic using fluorescence imaging for oral squamous cell carcinoma detection. (a) The diagnostic procedure in oral squamous cell carcinoma treatment. The fluorescent agent is topically administered to the patient. Due to the topical administration, the fluorescent signal can be detected immediately to assess the lesion and guide biopsy. Note that additional lesions otherwise missed by standard of care can be visualised. (b) Intraoperative margin assessment using ex vivo fluorescence imaging. Directly after excision, the margins of the excised specimen are scanned to identify tumour‐positive resection margins and enable immediate re‐resection

Fluorescence imaging relies on visualising fluorophores that are excited by specific wavelengths of light, depending on the excitation spectra. Subsequently, light is re‐emitted with less energy but with longer wavelengths. The penetration depth depends on the investigated wavelengths, ranging from a few hundred micrometres up to several millimetres, where a trade‐off exists between resolution and imaging depth due to photon scattering. The near‐infrared (NIR) spectrum of 700–1200 nm, the so‐called “optical window,” has advantages for in vivo FI as there are less photon scattering, less haemoglobin absorption and minimal tissue autofluorescence compared to the visual spectrum. Since OSCC is of epithelial origin, FI could be a well‐suited technique for tumour visualisation as well as being of added value in both diagnostics and treatment by providing information in a *real‐time* setting (Figure 1) (Hussain & Nguyen, 2014; Kossatz, Weber, & Reiner, 2017).

Whereas fluorescence in the visual spectrum can be detected by the human eye, fluorescence in the NIR spectrum requires a dedicated, highly sensitive camera system (e.g., charge‐coupled device camera with optical lenses and appropriate filters) as it is not visible to the human eye. Many FI systems have been evaluated to date, and some are commercially available (DSouza, Lin, Henderson, Samkoe, & Pogue, 2016).

Fluorescence contrast can be either generated by the visualisation of intrinsic fluorophores (e.g., NADH/FAD concentrations in autofluorescence imaging) (Solomon, Liu, Berezin, & Achilefu, 2011; Veld, Witjes, Sterenborg, & Roodenburg, 2005) or by the application of exogenous contrast agents (Gao et al., 2018; Kossatz et al., 2017; Rosenthal et al., 2015). Exogenous contrast agents can be divided further into non‐specific and targeted agents that can be administered topically or intravenously.

## 3 AUTOFLUORESCENCE IMAGING

Autofluorescence imaging (AFI) utilises the increase or loss off of intrinsic fluorophores for contrast generation. Typically, (pre‐)malignant lesions are associated with a loss of autofluorescence compared to benign oral mucosa and appear dark during imaging (Poh et al., 2006). AFI of the oral cavity has been pursued by a number of studies with several imaging devices, including the VELscope (LED Dental, Inc.). However, this method is considered of limited value for clinical decision‐making due to its low specificity (Macey et al., 2015) as many benign lesions also show loss of autofluorescence (Amirchaghmaghi et al., 2018). AFI optimisation through other techniques, such as diascopic fluorescence, might improve imaging, although their performance is debatable (Lalla, Matias, & Farah, 2016).

Thus, although AFI is easy to implement, it may not be the optimal tool for diagnostic use since the current diagnostic workup requires the ability to discriminate between malignant and benign lesions rather than malignant lesions and healthy tissue. AFI might, however, be suitable as an adjuvant diagnostic method to conventional oral examinations (Tiwari, Kujan, & Farah, 2019).

## 4 NON‐TARGETED EXOGENOUS FLUORESCENCE IMAGING

Non‐targeted fluorescent tracers are distributed through the vascular system to provide morphological and physiological information on the tissue of interest.

Indocyanine green (ICG) is one of the oldest clinically available fluorescent dyes. It has proven to be valuable for intraoperative imaging of tissue perfusion, for vascularisation and for sentinel lymph node mapping (Schaafsma et al., 2011). ICG accumulates in the tumour via the enhanced permeability and retention (EPR) effect (Maeda, Nakamura, & Fang, 2013), which exploits the increased vascularisation and decreased lymphatic drainage typically seen in tumour tissue. To date, only one study reported on the use of ICG in OSCC (Stubbs et al., 2019). In this study, patients received intravenous administration of ICG one day prior to surgery. Immediately after tumour excision, images were obtained of the excised specimen and the wound bed to assess the resection margins. Although an ICG signal was detected in 86% of the tumours, the lack in specificity limits the applicability of ICG for intraoperative resection margin assessment in OSCC. Moreover, as the working mechanism of ICG does not achieve a ligand–receptor binding, ICG is cleared during histopathological processing and thus cannot be used for ex vivo analysis and validation with histopathology. Another non‐targeted exogenous tracer, 5‐aminolevulinic acid (5‐ALA), has been proposed as an aid in the detection of oral cancer. Although 5‐ALA is, in itself, not fluorescent, it is a precursor of protoporphyrin IX (PPIX) in the haem synthesis pathway, which does have photosensitising abilities. Exogenous administration of 5‐ALA leads to a selective accumulation of PPIX in mitochondria of tumour cells. 5‐ALA has been administered topically and intravenously for diagnostic purposes in oral cancer. Compared to the gold standard, 5‐ALA is up to 99% sensitive but has limited specificity (60%) (Leunig et al., 2000). Specificity could be improved by up to 90% using an endoscopic system, although this compromises the wide‐field possibilities of FI (Zheng, Soo, Sivanandan, & Olivo, 2002). Nevertheless, studies report on the detection of malignant lesions which were missed by the standard of care (Leunig et al., 2000).

## 5 TARGETED FLUORESCENCE IMAGING

Over the last few decades, the interest in FI has shifted to imaging compounds targeting tumour‐specific biomarkers to improve tumour specificity. These include both receptors or proteins that are overexpressed in tumour cells or the tumour microenvironment and alterations in the metabolic activity such as tumour acidosis.

Topical administration of an imaging agent targeting the altered glycosylation in cancer cells has been studied using wheat germ agglutinin conjugated with fluorescein isothiocyanate (WGA‐FITC). In one study investigating WGA‐FITC in patients with OSCC, all 64 malignant lesions were detected, but specificity was limited (82%) due to signal detection in benign inflammatory lesions (Baeten et al., 2018). Another group is pursuing the detection of OSCC by topical administration of poly(ADP‐ribose) polymerase 1 (PARP1) inhibitors conjugated to fluorescent dye molecules (PARPi‐FL) (Kossatz et al., 2016, 2019). A preclinical study shows that PARP1 is significantly overexpressed in OSCC and that PARPi‐FL accumulates with high specificity in OSCC, also immediately upon topical administration (Kossatz et al., 2017). Currently, a clinical trial is being undertaken to evaluate the topical administration of PARPi‐FL in patients with OSCC for diagnosis enhancement (NCT03085147).

Intravenous administration of fluorescent imaging agents generally increases the tracer homogeneity in the target tissue, therefore providing better contrast compared to topical tracers. Ideally, these agents should be broadly applicable, adequately distributed throughout the body and target‐specific activatable to enhance the target‐to‐background ratio (TBR). This was partly achieved in the first‐in‐human targeted fluorescence study that used folate‐FITc for tumour detection during ovarian cancer surgery in 2011 (van Dam et al., 2011). The few current clinical trials of clinically available monoclonal antibodies labelled with a fluorescent dye targeting the epidermal growth factor receptor (EGFR) found overexpression in >90% of the patients with OSCC. The Rosenthal et al. group reported extensively on EGFR‐targeted fluorescence‐guided surgery using cetuximab‐IRDye800CW and panitumumab‐IRDye800CW (Warram et al., 2016). In their first study with 12 OSCC patients, the cetuximab‐IRDye800CW tracer was deemed safe since no grade 2 or higher adverse events were observed. The tumour was adequately differentiable from normal tissue, with TBRs as high as 5.2. Subsequently, the focus was shifted to panitumumab‐IRDye800CW, as this tracer had an improved safety profile in higher dose groups. The agent was administered 2–5 days prior to surgery. During surgery, the tumour tissue was delineated with an open‐field FI camera system which, on being applied at several moments, guided the resection and resulted in adequate TBRs in vivo. This approach also enabled the detection of satellite lesions which were missed by the standard of care. Immediately after tumour excision, the margins of the wound bed and the freshly excised specimen were imaged and assessed, which opens up the possibility for an immediate re‐resection to ensure radical excision. In their study cohort of 14 patients, the surgical decision could be altered in 21% of the cases on using FI. One OSCC satellite lesion was detected prior to surgery outside the planned surgical excision, and a close resection margin was identified on the excised specimen directly after resection. In another study, tumour detection within 5 mm of the resection margin was 95% sensitive and 89% specific when applying FI on the freshly excised specimen. Immediate detection of these tumour‐positive resection margins might enable immediate correction and improve surgical outcome (Keulen, Nishio, et al., 2019).

These phase I clinical trials that report on fluorescence‐guided surgery have shown accurate delineation of tumours that could assist in surgical resection margin assessment. Phase II and III trials need to commence to determine the impact of FI on surgical outcomes and prognosis, that is local recurrence and (disease‐free) survival.

## 6 IMPLEMENTING FLUORESCENCE IMAGING IN THE CLINIC

Tumour detection at an early stage and complete tumour removal during surgery are the most important steps towards adequate OSCC treatment. FI is a reliable and safe method to visualise biomarkers associated with carcinogenesis. Currently, the general consensus is that the use of targeted exogenous contrast holds the greatest potential for clinical use, since high tumour specificity can be obtained, compared to autofluorescence and non‐targeted tracers. Whereas photon scattering hampers the clinical use of FI for the detection of subsurface disease, it does not diminish the value of FI in the oral cavity since OSCC is of epithelial origin. Clinical demands for FI differ between diagnosis and surgery but require a maximum penetration depth of up to 5 mm for margin assessment.

For diagnostic purposes, topically applied tracers are preferred over intravenous tracers for several reasons. First, intravenous administration causes patient burden, which might not be justified considering the majority of the investigated lesions will be benign. Moreover, the interval needed between intravenous administration and imaging is logistically challenging for using FI as a screening method. Topically applied imaging agents do not come with these characteristics and can be easily implied by primary caregivers. Administration of fluorescence contrast as a rinse solution enables real‐time guidance of biopsies. Preferably, FI in the visible spectrum is performed as this enables cheap and straightforward assessment of the oral cavity, and no additional (expensive) camera systems are required. The additional penetration depth NIR can offer (i.e., penetration depth) is of limited value in diagnosis since OSCC is of epithelial origin.

For surgical use, intravenous administration of the imaging agent is preferred since it provides optimal distribution in the target tissue required for accurate tumour delineation. Here, opposed to the diagnostic setting, imaging in the NIR spectrum is crucial, as margin assessment up to a depth of 5 mm is needed for intraoperative decision‐making. During surgery, FI can be used in vivo to determine the surgical plan by evaluating the entire surgical field for the presence of multifocal tumour loci and can aid in tumour delineation. To enable accurate assessment of the resection margins, analysis of the freshly excised specimen (i.e., when the patient is still anaesthetised), denominated as imaging‐assisted pathology, is a more robust alternative compared to in vivo assessment for several reasons. First, in vivo FI cannot assess the deep resection margin due to the limited penetration depth. Also, FI of the wound bed is affected by non‐specific optical phenomena (e.g., blood or coagulation artefacts). Last, analysing the specimen outside the sterile working field is less susceptible to external factors that influence imaging results such as the angle and distance of imaging and other light sources in the operating room.

Notwithstanding the successful application of imaging‐assisted pathology in highlighting areas at risk of tumour‐positive resection margins (van Keulen, van den Berg, et al., 2019), the microscopic assessment of the resection margins might be challenging due to the spatial resolution of FI. We advocate the use of wide‐field FI to highlight suspect areas (i.e., red‐flag technique) together with complementary high‐resolution imaging techniques to assess particular areas in more detail, such as spectroscopy‐based techniques or high‐resolution microscopy (Glaser et al., 2017), which fall outside the scope of the current review.

## 7 CONCLUDING REMARKS

Despite the many efforts to improve patient prognosis in OSCC, survival rates have only improved slightly over the last decade. This emphasises the need for tools that improve tumour detection for both diagnoses and surgical guidance during resections. The current literature shows encouraging data on the use of FI for the diagnosis and treatment of OSCC. Several clinical phase I studies report increased detection rates when using FI compared to standard care, both preoperatively and during surgery, where targeted imaging agents show better results compared to non‐targeted and AFI methods due to increased specificity. The high tumour specificity of targeted imaging is an essential parameter, both for discriminating between malignant and benign lesions during diagnosis and tumour delineation during surgery. However, important issues need to be explored further in upcoming clinical trials, such as the positive and negative predictive factors, the ease of use and the interpretation of imaging results by (non‐expert) clinicians, primarily since the optical properties of tissue might influence the imaging results. Setting‐specific requirements such as dosing‐to‐imaging intervals and tissue penetration define different requirements for an optimal imaging agent in the diagnostic and surgical setting. Ideally, integrating FI in the clinical decision‐making progress could result in a dynamic and flexible process, providing the clinician with valuable additional information during the multiple clinical steps. Presumably, FI will be used together with complementary imaging techniques that have a limited field of view but can provide greater detection sensitivity, higher resolution or quantitative measurements (e.g., spectroscopy methods).

Supported by the feasibility studies' findings, phase II/III clinical trials now need to commence to investigate the clinical benefit of FI. A combined effort by several renowned imaging groups might be essential for this relatively small field and might lead to the swift implementation of FI in the clinic.

## AUTHOR CONTRIBUTION

JV, FJV and MJHW designed the manuscript. JV, JGW, FJV and MJHW wrote the manuscript. JGW designed the figure.
